# Sequence specific visual detection of LAMP reactions by addition of cationic polymers

**DOI:** 10.1186/1472-6750-6-3

**Published:** 2006-01-10

**Authors:** Yasuyoshi Mori, Tsuyoshi Hirano, Tsugunori Notomi

**Affiliations:** 1 Eiken Chemical Co., Ltd. 1381-3 Shimoishigami, Ohtawara, Tochigi, 324-0036, Japan

## Abstract

**Background:**

Development of a practical gene point-of-care testing device (g-POCT device) requires innovative detection methods for demonstrating the results of the gene amplification reaction without the use of expensive equipment. We have studied a new method for the sequence-specific visual detection of minute amounts of nucleic acids using precipitation reaction by addition of cationic polymers to amplicons of Loop mediated isothermal Amplification (LAMP).

**Results:**

Oligo DNA probes labeled with different fluorescent dyes were prepared for multiple nucleic acid templates, and the templates were amplified by the LAMP reactions under the existence of the probes. At completion of the LAMP reaction, an optimal amount of low molecular weight polyethylenimine (PEI) was added, resulting in the precipitation of the insoluble LAMP amplicon-PEI complex. The fluorescently labeled Oligo DNA probes hybridized to the LAMP product were incorporated into the precipitation, and the precipitate emitted fluorescence corresponding to the amplified nucleic acid templates. The color of emitted fluorescence can be detected easily by naked eye on a conventional UV illuminator.

**Conclusion:**

The presence or absence of minute amount of nucleic acid templates could be detected in a simple manner through visual assessment for the color of the LAMP amplicon-PEI complex precipitate. We conclude that this detection method may facilitate development of small and simple g-POCT device.

## Background

**Figure 1:**
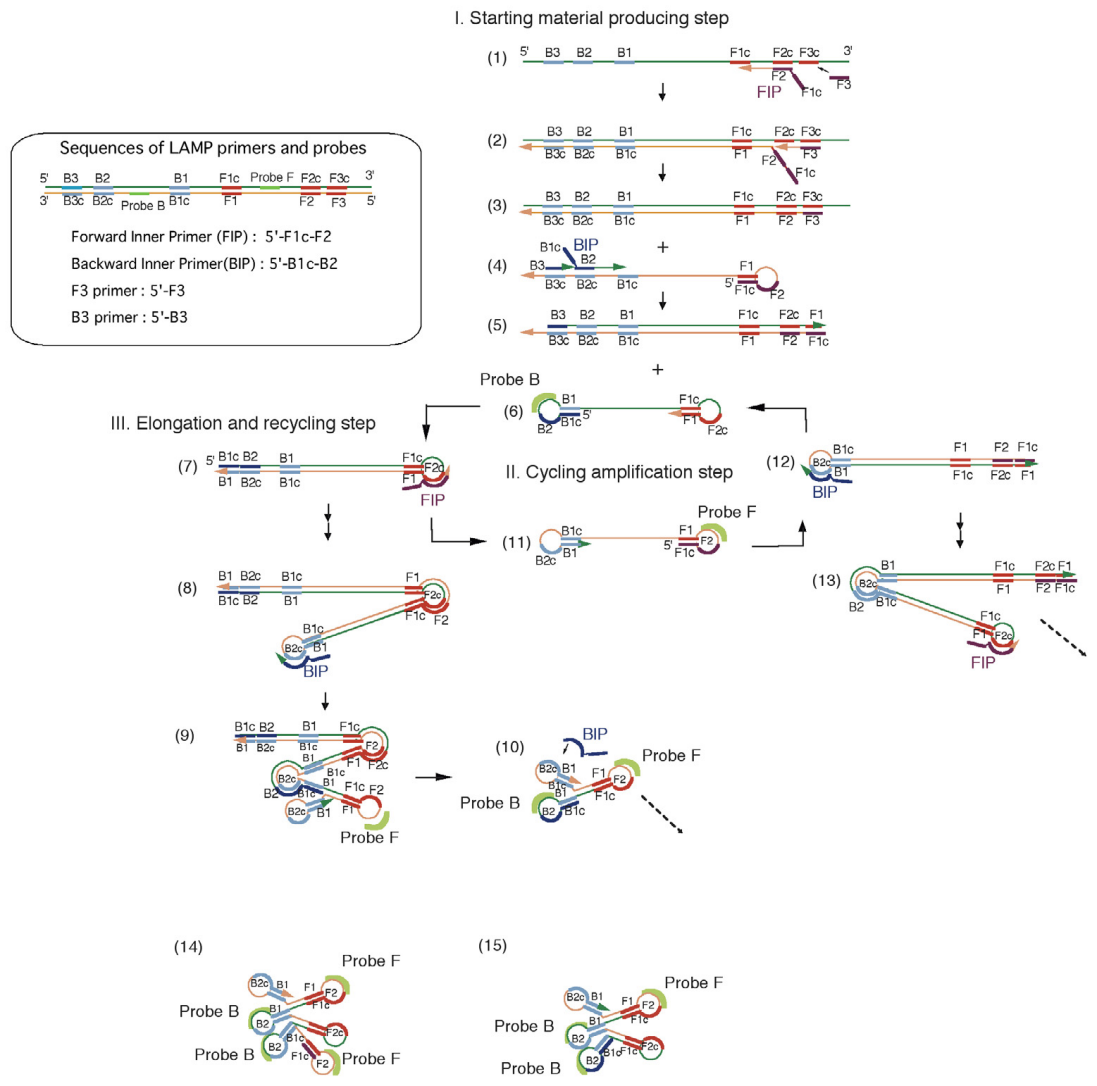
**Pattern diagram of LAMP reaction and hybridization of fluorescently labeled oligo DNA probes**. The LAMP reaction takes place in three steps (starting material production step, cycling amplification step, and elongation and recycling step) by the primers depicted in the enclosure. In the starting material production step, the starting material (6) is generated by primers (forward inner primer (FIP) and backward inner primer (BIP)). A complementary strand (11) of the starting material (6) is synthesized from the starting material (6) by a reaction that uses itself as a template and by a reaction from an FIP annealed to the loop segment, thus making up the cycle amplification step. During this step, probes (probe F and probe B, respectively) designed for the region between the F1 and F2 region or the B1 and B2 region can hybridize to the loop segment. As the cycle reaction progresses, an elongation and recycling step takes place, during which elongated products (8, 13, etc.) with an inverted repeat structure are generated. Numbers 14 and 15, which have a cauliflower structure, are also generated. They have many loop structures to which probes can hybridize.

Loop-mediated isothermal amplification (LAMP) is a unique gene amplification method in which DNA can be isothermally amplified using only one enzyme [1-3]. Since the advent of the LAMP method, many researchers have been engaged in basic research from a variety of perspectives. As a result, it is currently being put to practical use in the reagents for detecting various pathogens such as SARS [4] and the West Nile virus [5] and reagents for identifying the sex of fertilized eggs in cow *in vitro *fertilization [6]. Furthermore, LAMP is a gene amplification method with a variety of characteristics and applications in a wide range of fields, including SNP typing [7] and quantification of template DNA [8]. In particular, LAMP is considered to be effective as a gene amplification method for use in gene point-of-care testing (g-POCT) devices, which are used for simple genetic testing whenever and wherever necessary. First, since LAMP can amplify genes isothermally, the amplification reaction can be carried out with a simple heater. There is no need for the special device used for polymerase chain reaction (PCR) to rapidly control the temperature [3]. Next, a large amount of DNA (10–30 μg/25 μl) can be synthesized in a short time (15–60 min) while maintaining high specificity. This characteristic greatly facilitates detection of the LAMP reaction [9]. Moreover, since the LAMP reaction progresses by generating a characteristic stem-loop structure, LAMP products have a single-stranded segment in the molecule (loop segment; see Figure 1 and reference No. 1). By using oligo DNA probes designed to recognize the sequence of the single-stranded segment, it is possible to carry out hybridization assay without performing heat denaturation after amplification. This means that all processes, from the amplification reaction to the detection reaction, can be carried out completely isothermally.

If these characteristics of the LAMP method are used effectively, we believe it will be possible to develop simple genetic testing devices that have not been realized yet despite a strong awareness of their necessity, in a wide range of fields, including infectious disease testing, food inspection, and environmental testing. The key to developing such simple devices will be figuring out how to simply and clearly present the final amplification results. The objective of this research is to establish new techniques for sequence-specific visual detection of amplification results by means of the LAMP method. To that end, we made use of a reaction that has been known for a long time, i.e., cationic polymers like polyamines form an insoluble complex with DNA [10]. It is well known that one of such the polyamine, polyethylenimine (PEI), strongly interacts with DNA. PEI is widely used as a nucleic acid precipitant for nucleic acid purification [11] and as an *in vivo *and *in vitro *non-viral vector [12,13]. We discovered that an insoluble PEI-LAMP product complex was generated under certain optimized conditions when PEI was added to LAMP reaction solution. Using this precipitation titration, we investigated whether it was possible to perform bound/free separation of fluorescently labeled probes. In this paper, we report the novel visual detection methods of the presence of sequences of HBV and HCV cloned to plasmid as a model experiment using this method and the results of an investigation of the basic reaction conditions required to achieve this.

## Results

### Mechanism of LAMP reaction and hybridization of probe

The primers used for LAMP reaction is schematically depicted in enclosure of Figure 1. Forward Inner Primer (FIP) consists of F2 and the complementary sequence of F1, and Backward Inner Primer (BIP) contains of B2 and the complementary sequence of B1 when each sequences (F1, F2, B1, and B2) are defined on the template sequence as shown in Figure 1. In some references such as Notomi et al. [1] or Parida et al. [5], a spacer of few thymidines was inserted between F1c or B1c and F2 or B2 in the inner primer (FIP or BIP) so that one and two thymidine spacers were inserted in FIP and BIP of HBV, respectively. However, the spacer was not used in this study because the LAMP reaction can progress with the use of inner primers without the spacer as shown by Hong et al. [4], Hirayama et al. [6], and Iwasaki et al. [7]. The LAMP reaction takes place isothermally in the three steps shown in Figure 1, i.e., starting material production step, cycling amplification step, and elongation and recycling step, by using of polymerase with strand displacement activity. First, the starting structure (structure 6) is generated from the template nucleic acid in the starting material production step. Next, the starting structure becomes structure 7 with a stem-loop structure by self-primed DNA synthesis. When the forward inner primer is hybridized to the loop segment and strand displacement synthesis takes place, structure 11, which is a complementary strand of structure 6, is generated. This means that an auto cycle reaction was established between structure 6 and structure 11. In addition, products bound by an inverted repeat with two amplified regions, like structures 8 and 13, are generated in association with the auto cycle reaction (cycling amplification step). Then, with these structures as starting points, products elongated to a length of several kbp and products with complex structures with cauliflower-like structures (14, 15) are ultimately generated.

Since the LAMP products have a loop structure, oligo DNA probes (green arcs in the figure) in the reaction solution can sequentially hybridize to products as the LAMP reaction proceeds. The cauliflower structures, in particular, contain two or more loops to which the probes can hybridize. This characteristic of the LAMP reaction plays an important role in the detection method described here.

### Sequence-specific visualization of LAMP amplicons and it's estimated mechanism

**Figure 2:**
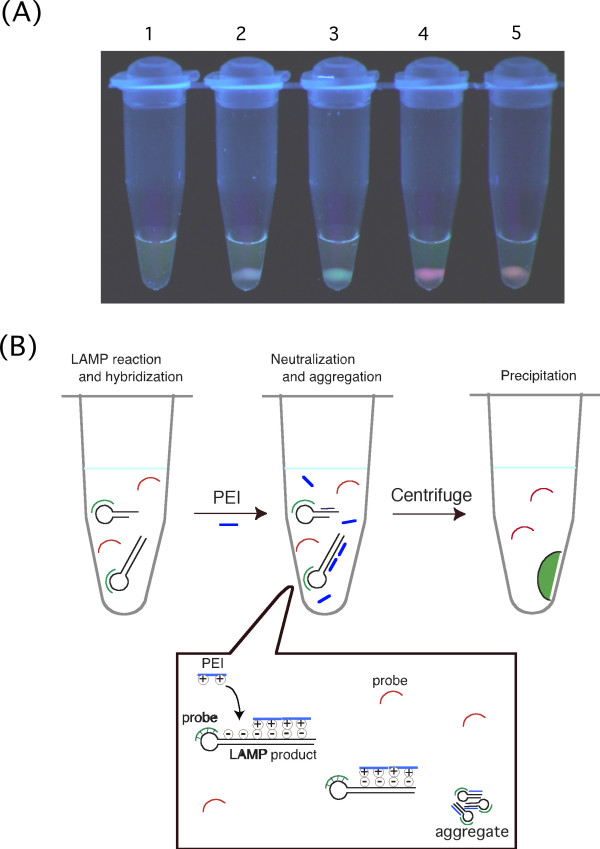
**Sequence-specific visual detection method that utilizes precipitation titration of LAMP products by adding PEI**. (A) Results of sequence-specific visual detection after adding PEI to LAMP reaction solution. After LAMP reaction in the presence of both FITC-labeled HBV probes and ROX-labeled HCV probes followed by addition of the prescribed amount (0.2 μmol as monomer) of PEI (Mw = 600), it was centrifuged for several seconds using a desk-top, low-speed centrifuge. The tube was then visually observed as is on a UV illuminator (365 nm). It was possible to differentiate the LAMP reaction by visualizing the presence of precipitate fluorescence and the color of the fluorescence. 1, LAMP reaction negative. 2, When LAMP reaction with PSA amplification (unrelated LAMP reaction) occurred. 3, When it contained HBV template nucleic acid. 4, When it contained HCV template nucleic acid. 5, When it contained both HBV and HCV template nucleic acids. (B) Diagram of principle of sequence-specific visual detection method that utilizes precipitation titration of LAMP products by adding PEI. First, a LAMP reaction is carried out using a LAMP primer set for two types of template nucleic acid and fluorescently labeled probes, which can hybridize to loop segments of each LAMP products. When a LAMP reaction corresponding to a certain fluorescently labeled probe progresses, the probe will sequentially hybridize to the loop segment generated during the reaction. On the other hand, an unrelated probe remains free in the solution. When an optimized amount of PEI is added after reaction for a set length of time, the positive charge of PEI neutralizes the negative charge of the DNA to form an insoluble LAMP product-PEI complex. At this stage, fluorescently labeled probes hybridized to LAMP products are taken up by the LAMP product-PEI complex together with the LAMP products. Since most of the PEI added is used for formation of the LAMP product-PEI complex, free oligo DNA probes cannot form a complex with PEI. When the generated insoluble complex is pelletized by centrifugation and the pellet is irradiated with excitation light, the labeled fluorescent dye hybridized to LAMP products produces fluorescence.

Plasmid DNA cloned with HBV or HCV sequence was added to a LAMP reaction solution containing both HBV primers and the probe (FITC-labeled) and HCV primers and the probe (ROX-labeled) and amplified, after which low molecular weight PEI (Mw = 600; 0.2 μmol as a monomer) was added. As shown in Figure 2A, precipitate emitting green fluorescence characteristic of FITC was obtained in LAMP reaction solution containing the HBV template, precipitate emitting red fluorescence characteristic of ROX was obtained in LAMP reaction solution containing the HCV template, and precipitate emitting a color (orange) that was a combination of FITC green and ROX red fluorescence was obtained in LAMP reaction solution containing both templates. They could be observed using an ordinary UV illuminator or UV-LED (light emitted diode). When LAMP products (lambda DNA) not related to HBV and HCV were present, precipitate with no fluorescence was obtained, and visible precipitate was not generated in a sample not containing a template (LAMP reaction negative). This means that it was possible to assess whether the HBV template nucleic acid was in the reaction solution, HCV template nucleic acid was in the reaction solution, or both were in the reaction solution by visualizing the fluorescent color of the precipitate. The LAMP method is a nucleic acid amplification method that is so sensitive that it is possible to create an amplification reaction from only six copies of template nucleic acid [1]. Therefore, a combination of LAMP amplification and this detection method makes possible sequence-specific visual presentation of the presence of trace amounts of nucleic acid, i.e., only several copies, found in a sample.

A model that represents the principle of this method is shown in Figure 2B. Oligo DNA probes labeled with fluorescent dye are hybridized to a specific LAMP product. When an optimized amount of low-molecular-weight PEI (Mw = 600) is then added, the positive charge of PEI neutralizes the negative charge of the DNA, which results in formation of an insoluble DNA-PEI complex. When this solution is left to stand for a few minutes or it is centrifuged with a small, desk-top centrifuge for a few seconds, the complex is deposited at the bottom of the tube. When the precipitate is observed on an illuminator (365 nm), the fluorescence of the dye from the probe taken up by the precipitate is visualized. On the other hand, probes unrelated to the sequence of the amplified LAMP product are not taken up by the LAMP-PEI complex because they are not hybridized to the LAMP product. When the molecular weight of the PEI used is small, oligo DNA probes and PEI cannot interact sufficiently to form an insoluble complex. Therefore, unrelated probes remain in the supernatant. Since the fluorescent probes in the supernatant are dispersed, the fluorescence cannot be visualized. Consequentially, bound/free separation of labeled oligo DNA is achieved as a result of insolubilization by PEI of LAMP products.

### Confirmation of the mechanism of the sequence-specific visualization method

**Figure 3:**
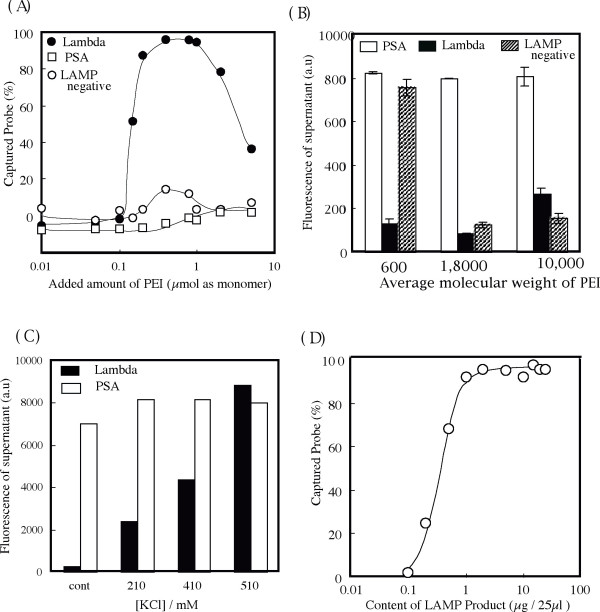
**Precipitation titration of LAMP products by addition of PEI**. (A) Effect of amount of PEI on sequence-specific incorporation of ROX-labeled lambda DNA recognition probes by DNA-PEI complex (Mw of PEI is 600). When 0.2 μmol to 1.0 μmol of PEI was added as a monomer, almost 100% of labeled probes hybridized to the LAMP products for lambda DNA was taken up by the DNA-PEI complex. On the other hand, when 0.4 μmol to 0.8 μmol of PEI was added as a monomer to a reaction solution in which an amplification reaction did not take place, a small amount (<20%) of labeled probes precipitated. Precipitation of this nonspecific probe did not take place when unrelated LAMP products (PSA) were present. (B) Effect of the Mw of PEI on sequence-specific incorporation of ROX-labeled lambda DNA recognition probes by DNA-PEI complex. When PEI with Mw 600 was used, the fluorescence intensity of supernatant decreased only when LAMP product for lambda DNA was present. As the Mw of the PEI used increased from 1,800 to 10,000, the fluorescence intensity of the supernatant decreased in the absence of a LAMP reaction since the formation of insoluble PEI-oligo DNA probe complex occurred. (C) Effect of amount of KCI on sequence-specific incorporation of ROX-labeled lambda DNA recognition probes by LAMP product-PEI (Mw = 600) complex. Normal LAMP reaction solution (control) contains 10 mM of KCI. Since formation of the PEI-LAMP product complex was inhibited by an increase in the amount of KCI added, the fluorescence intensity of supernatant increased regardless of the sequence of the LAMP product. (D) Effect of amount of LAMP product for lambda DNA on sequence-specific incorporation of ROX-labeled lambda DNA recognition probe by LAMP product-PEI complex (Mw of PEI = 600). Even when the amount of LAMP product was 1 μg per 25 μL, almost 100% of labeled probes (1 pmol) was taken up by PEI-DNA complex.

The following experiments were conducted to confirm this principle. First, we investigated the effect of the added amount of PEI on this precipitation titration (Figure 3A). Oligo DNA probes for lambda DNA were captured in the precipitate when 0.2 to 1 μmol of PEI was added to 25 μL of LAMP reaction solution for lambda DNA. If the amount of PEI added is too high or too low relative to the optimal range, the PEI-DNA complex precipitate is not formed, resulting in oligo DNA probes remaining in the solution. This phenomenon is characteristic in ionic interaction between cationic polymers and anionic polymers [14]. Namely, when the amount of the cationic polymer PEI is too low, the PEI-DNA complex becomes anionic. In contrast, when the amount of PEI is too high, the PEI-DNA complex becomes cationic. In both cases, the PEI-DNA complex is solubilized as a result. This characteristic, which is shown in Figure 3A, indicates that this precipitation titration is based on neutralization of the negative electric charge of the DNA by the cationic polymer PEI. In the case of LAMP negative, a small amount of free probe was deposited under certain conditions when 0.4 μmol to 0.8 μmol of PEI was added as a monomer (< 20%). However, the amount of precipitate in this case was so small that it was impossible to confirm it visually. On the other hand, when LAMP products (PSA) unrelated to the probe sequence were present, no free probe at all was deposited. This is because almost all of the PEI molecules added were consumed in the precipitation of unrelated LAMP products, an excess of which was present relative to the amount of probe. In other words, more reliable detection is achieved by first confirming whether the LAMP reaction has occurred by checking whether white DNA-PEI complex precipitate is generated as a result of addition of PEI and then determining for which nucleic acid template the LAMP reaction occurred based on the fluorescent color of the precipitate. Because of the above results, the amount of PEI added was fixed at 0.2 μmol for the following experiments in order to avoid generation of free probe precipitate as much as possible.

We investigated the effect of the Mw of PEI on this detection system (Figure 3B). When PEI with different Mw was added to the LAMP solution so that the amount per monomer of each was the same, we found that sufficient BF separation occurred if LAMP amplicons were present, even if the Mw of PEI increased up to 10,000. That is, the fluorescence intensity of the supernatant of LAMP solution with specific amplification (lambda DNA) is lower than that with unrelated amplification (PSA). As the Mw of PEI increased, however, almost all probes in LAMP reaction negative solution formed an insoluble PEI-oligo DNA complex. This result means that the LAMP reaction negative solution cannot be distinguished from the LAMP reaction positive solution, which successfully amplifies the targeting sequence if PEI with a high molecular weight is used. As was also observed in Figure 3A, when LAMP amplicons are present, almost all of the added PEI reacts with LAMP amplicons, so there is little opportunity for interaction with free oligo DNA probes, but when LAMP amplicons are not present, all of the added PEI interacts with oligo DNA probes. Under conditions where a large amount of PEI with a high molecular weight can strongly interact with oligo DNA, an undesirable insoluble PEI-oligo DNA probe complex forms because the molecular weight of PEI is high. Therefore, the average molecular weight of PEI in this detection system should be about 600. We conducted a similar experiment using spermine, which is a polyamine with a lower molecular weight. In that experiment, an adequate amount of insoluble complex was not generated compared with PEI of an average molecular weight of 600 under conditions used for the present research (data not shown). It is well known that spermine can also make DNA insoluble, as indicated in many other reports [10]. Therefore, optimal conditions in the case of spermine as a precipitant might exist, but since further investigation would have gone beyond the scope of this paper, no further investigation was carried out.

Insolubilization of LAMP products by PEI was inhibited by addition of an excessive amount of KCl to the LAMP reaction solution after amplification (Figure 3C). This was because excessive amounts of potassium ions and chloride ions inhibited the electrostatic interaction between DNA and PEI. This finding is further indication that this detection method is based on neutralization of the negative electric charge of DNA by the positive electric charge of PEI. The effect of ionic strength on the LAMP reaction has been investigated and found that the presence of 200 mM or more of KCl markedly delayed the LAMP reaction (data not shown). Therefore, it can be said that this detection method will function without trouble if the solution used has a composition that is optimized for the LAMP reaction.

We investigated the sensitivity of this detection method (Figure 3D). Almost all probes were taken up by the precipitate in the case of up to 1 μg of LAMP product. Moreover, we were able to visualize the fluorescence in the precipitate even if the specific LAMP product was 0.2 μg. We found that this detection system was sensitive enough as a detection system for visual assessment to be used in simple g-POCT devices. We can see from the results shown in Figure 3C that all of the 1 pmol of probe added hybridized to 1 μg (= 3 nmol nucleotide) of LAMP product. In other words, one molecule of probe bound to every 1,500 base pairs of LAMP products. The LAMP product is a mixture of products of several different sizes, with an average molecular size of 2 kbp [1]. This finding that one molecule of probe binds to every 1,500 base pairs reflects well the fundamental characteristic of this LAMP reaction.

## Discussion

The new detection method described above utilizes the unique nature of low-molecular-weight PEI, i.e., it cannot form an insoluble complex with a single-stranded anionic polymer with a low molecular weight such as an oligo DNA probe, but it can form an insoluble complex with DNA with a high molecular weight such as LAMP product. Until now, not much attention appears to have been paid to the complex consisting of oligo DNA and PEI with a molecular weight of 1,000 or lower, which was used in this study. This is probably because the interaction between low-molecular-weight DNA and oligo DNA is very weak and because its practical utility as a vector or nucleic acid precipitant is low. However, Kunath et al. reported that the complex formed by PEI (5 kDa) and plasmid DNA was more unstable than that formed by PEI (25 kDa) [15]. Osland and Kleppe reported that spemidine, which has a structure similar to that of PEI, formed an aggregate with double-stranded oligo DNA, but it did not form an aggregate with single-stranded DNA [10]. Drawing upon these results, the low-molecular-weight PEI used in this study is thought to interact with DNA with high selectivity to differences in DNA concentration and structure (number of strands and molecular weight). The fact that we took advantage of this nature of low-molecular-weight PEI as a nucleic acid precipitant for detection led to the establishment of this detection method.

This detection method effectively utilizes the characteristics of the LAMP method, i.e., a large amount of amplification product can be synthesized in a short time while maintaining high specificity. Since a large amount of amplification product is created by the LAMP reaction, precipitate of a size that can be easily confirmed with the eyes is generated when PEI is added to the LAMP reaction solution. Moreover, the fact that the amplification is highly efficient means that the amount of labeled probe for detection that can be added is large. As a result of these characteristics, the LAMP reaction followed by addition of PEI yields precipitate with a clear color and in a size that can be identified visually, as shown in the photograph in Figure 2. Furthermore, the fact that the amount of the amplification product is large means that the range of the optimum amount of PEI necessary to generate the insoluble complex is wide. Thus, a precise system for adding the PEI solution is not necessary. This will contribute to simplification of g-POCT devices that use this detection method. In the case of PCR, the most widely used gene amplification method, the amount of amplification product is usually 1/20 or less that of LAMP [9]. Consequently, in order to apply this method to PCR, a device for rapidly cycling the temperature to perform PCR, a high-luminance fluorogenic reagent, a system for washing unreacted probes, and a system for accurately dispensing PEI would be necessary. This would likely be an obstacle to putting to practical use a simple, inexpensive g-POCT device for genetic testing.

There are several ways to make LAMP products insoluble besides addition of a cationic polymer like PEI. We tried carrying out the BF separation using chilled ethanol. Since the addition of chilled ethanol lowered the temperature of the LAMP solution, there was a strong tendency for nonspecific probes to weakly hybridize to LAMP products and precipitate (data not shown). Moreover, isopropanol and PEG are believed to be inferior to a PEI solution because it is necessary to add several times the amount of LAMP reaction solution and they are not as easy to handle. Therefore, we believe that low-molecular-weight PEI, which is efficiently insolubilizes amplicons with only a small amount, is the best option for high BF separation, as was shown in this study.

If the 5' end of the inner primer is fluorescently labelled, the LAMP product should be visible as in the current study. However, visualization using inner primers fluorescently labelled at the 5' end is not preferred, because the possibility of false positives from self-extension of the labeled primer cannot be excluded. There is no risk of false positives with the oligo DNA probes fluorescently labeled at the 3' end as in this study, so that highly accurate genetic testing can be established.

It is necessary to add PEI to the LAMP reaction solution after the LAMP reaction takes place since PEI strongly inhibits the LAMP reaction. However, opening the reaction tube after amplification is generally avoided to prevent carry-over contamination. Therefore, development of a technique for adding PEI in a closed system is needed to put this method to practical use. Some possible solutions could be to apply PEI to the lid of the reaction tube beforehand and turn the reaction tube upside down after LAMP amplification or use wax that responds to heat. Furthermore, we should be able to use technology like micro-Total Analysis system or Lab-on-a-Chip, for which much research is being conducted in recent years, to solve this problem.

## Conclusion

We established an extremely simple method for visually detecting LAMP products in a sequence-specific manner by simply adding a small amount of low-molecular-weight PEI to the LAMP reaction solution. The biggest feature of this technique is the ability to visually present sequence information of amplicons without using an expensive source of light or a detector. In contrast, conventional genetic tests require expensive reagents, complex and skillful manipulation, and large devices equipped with an expensive optical system. These are the main reasons why genetic testing is kept within the walls of specific institutions or university laboratories with special equipment and trained engineers. The combination of the LAMP method and the new detection method described here can overcome several factors that have been preventing true practical application of super-simple g-POCT devices for genetic testing. If a simple, inexpensive g-POCT device that is small and light enough to be held in one hand and whose main components are disposable can be developed, the day when parents will be able to genetically test for a pathogen while sitting next to the bed of their child who has a fever will not be far off [16].

## Methods

### Preparation of template DNA

Viral DNA of the hepatitis B virus (GenBank accession number: V00867) digested with BamHI was cloned to pBR322. Reverse transcriptase PCR was conducted for HCV viral RNA (GenBank accession number: AB031663) from patient serum purified using a QIAmp viral RNA Mini kit (Qiagen K.K.) with the PCR primers designed to the sequence of 5' non-coding region and core. The PCR product was cloned to pBR322 according to the established method. Concentrations of the respective plasmids obtained were determined by 260-nm spectrophotometer (Ultrospec 20000, Pharmacia Biotech). The template DNA solutions for LAMP reactions were prepared by serial dilution of the plasmid solutions and purchased lambda DNA solution (Takara Bio, Inc.) with Tris-HCl buffer (10 mM, pH8.0) until it contained 1,000 copies/5 μl.

### LAMP reaction and oligo DNA probes

The LAMP reaction was carried out on a scale of 25 μl based on reference 1. Briefly, a forward inner primer (FIP) and a backward inner primer (BIP) at a final concentration of 1.6 μM, F3 and B3 primers at a final concentration of 0.2 μM, fluorescently labeled probe at a final concentration of 40 nM (1 pmol/25 μl), Tris-HCl buffer at a final concentration of 20 mM, MgSO_4 _at a final concentration of 8 mM, dNTP at a final concentration of 5.6 mM, KCI and (NH_4_)_2_SO_4 _at a final concentration of 10 mM, betaine at a final concentration of 0.8 M, and Bst polymerase (New England BioLabs, Inc.) at a final concentration of 8 U were mixed, and 5 μL of template solution was added to make 25 μl, which was then heated for 30 min at 62°C. For simultaneous detection of HBV and HCV, 2.5 μl of each template solution was added.

The LAMP primers (Sigma Genosis Japan K.K., HPLC purification grade) were as follows. The hyphens were added between F1c or B1c and F2 or B2.

#### HBV

FIP:CCTGCTGCTATGCCTCATCTTCTT-T-GACAAACGGGCAACATACCTT

BIP:GATAAAACGCCGCAGACACATCC-TT-CCAACCTCTTGTCCTCCAA

F3: GGTGGTTGATGTTCCTGGA

B3: CAAAATTCGCAGTCCCCAAC

(FIP and BIP have one and two thymidine spacer, respectively)

#### HCV

FIP: CCGCAAGACTGCTAGCCGAG-GCAAGCACCCTATCAGGC

BIP: GGTTKATCCAAGAAAGGACCCAGTC-GCCATAGTGGTCTGCGGA

F3: CATGGTGCACGGTCTACG

B3: GGCGTTAGTATGAGTGTCGTAC

(K in BIP means G or T)

#### Lambda DNA

FIP: TGAAAATTCCCCTAATTCGATGA-GGTCGGCGCATAGCTGATAACAAT

BIP: TCCCCTCAGAACATAACATAGTAATGCGGTAA-GTCGCATAAAAACCATTC

F3: GCTGATCGGCAAGGTGTTCT

B3: GCTTATCTTTCCCTTTATTTTTGC

The LAMP reaction for Lambda DNA was used as a LAMP reaction for a basic investigation to clarify the fundamental characteristics of the reaction that occurs between the LAMP product and PEI. A well-established LAMP product [1] for mRNA of prostate-specific antigen (PSA) was used as a LAMP reaction product unrelated to the three LAMP reaction products above. A solution was prepared by serially diluting a LAMP reaction solution with a known concentration (28 μg DNA/25 μl) using LAMP reaction buffer in order to systematically investigate the reaction between the LAMP product and PEI. The concentration of DNA synthesized by the LAMP reaction was determined according to the attached protocol using a PicoGreen dsDNA Quantitation Kit (Molecular Probes, Inc.). The spectrofluorophotometer used was the RF-5000 spectrofluorophotometer (Shimadzu Scientific Instruments, Inc.).

The loop segments within LAMP products, i.e., sequences complementary to sequences between either F1 and F2 or B1 and B2, were used as probes for detection, as shown in Fig. 1. The probes were designed such that the sequences between F1c or B1c and F2c or B2c had a melting temperature (Tm) of 1 to 5°C lower than LAMP reaction temperature (62°C). All fluorescently labeled oligo DNA probes, which were HPLC purification grade, were purchased from Sigma Genosis Japan KK. The sequences of the probe used were as follows. The numbers in parentheses indicate the length and melting temperature (Tm) from supplier's information.

HBV (probe B): 5'- AGCGATAGCCAGGACAAA -FITC (18mer, 61.0°C)

HCV (probe F): 5'- TTGGGTTGCGAAAGG-ROX (15mer, 59.2°C)

Lambda (probe B): 5'- ATGGATTGAATTATGAAGAATG -ROX (22mer, 57.0°C)

### Preparation of PEI solutions

Commercially available PEI (Wako Pure Chemical Industries, Ltd.) was used without further purification. The average molecular weight of PEI used was 600, 1,800, and 10,000. In this study, the concentration of the PEI aqueous solution is expressed as the concentration of the monomer unit (-C_2_H_5_N-, 46 g/monomer). The PEI stock solution (2.0 mol/l) was prepared by dissolving 4.6 g of PEI in 50 mL of deionized distilled water in a graduated cylinder. At the time of use, the stock solution was diluted with water to the desired concentration.

### Sequence-specific detection of LAMP amplicons

White precipitate of magnesium pyrophosphate forms in the LAMP reaction solution as a by-product of the amplification reaction [5]. Therefore, after amplification, we first precipitated the magnesium pyrophosphate to the bottom of the reaction tube by centrifugating the LAMP reaction solution for 10 seconds using a small desk-top centrifuge (6,000 rpm). Then, 4 μl of PEI solution adjusted to the desired concentration was added to the supernatant to form an insoluble DNA-cationic polymeric polymer complex at room temperature. A pellet was formed by immediately centrifuging it for 10 seconds using the same centrifuge. A fluorescent image of the pellet was photographed with a fluorescence microscope (VB-G05, Keyence Corporation) equipped with a Handy UV Lamp (wavelength: 365 nm; Vilber Lourmat) and a UV blocking filter (NEO Dynamic L-400, MARUMI Optical Co., Ltd.). Since the magnesium pyrophosphate precipitate was nonfluorescent, it had no effect at all on visualization of pellet fluorescence. To investigate the effect of ionic strength on precipitation titration, aqueous KCl was added to the reaction solution after the LAMP reaction so that it was the prescribed concentration.

After centrifugation, 20 μl of supernatant of the PEI-DNA complex solution was aliquoted to a 384-well assay plate (Corning, Inc.) for fluorometry using a fluorescence plate leader (Polarion; Tecan Japan Co., Ltd.). The percentage of probes captured in the precipitate of the PEI-LAMP product complex was calculated according to the following formula based on the obtained results.

Captured probe (%) = (I_O _- I_p_) × 100/I_O_

Where I_O _and I_p _are fluorescence intensity of with and without addition of PEI solutions, respectively.

## List of abbreviations

LAMP – Loop mediated isothermal amplification, PEI- polyethylenimine, FIP – forward inner primer, BIP – backward inner primer, g-POCT – gene point of care testing, PCR – polymerase chain reaction, Mw – average molecular weight, BF separation – bound/free separation

## Authors' contributions

YM developed idea, performed the experiments, and drafted the manuscript. TH carried out the experimental work. TN envisioned and supervised all the studies. All authors read and approved the final manuscript.

## References

[B1] Notomi T, Okayama H, Masubuchi H, Yonekawa T, Watanabe K, Amino N, Hase T (2000). Loop-mediated isothermal amplification of DNA. Nucleic Acids Res.

[B2] Notomi T, Nagamine K, Mori Y, Kanda H, Demidov VV, Broude NE (2004). DNA amplification: Loop-mediated Isothermal Amplification (LAMP) of DNA Analysis. DNA Amplification.

[B3] Nagamine K, Watanabe K, Ohtsuka K, Hase T, Notomi T (2001). Loop-mediated isothermal amplification reaction using a nondenatured template. Clin Chem.

[B4] Hong TC, Mai QL, Cuong DV, Parida M, Minekawa H, Notomi T, Hasebe F, Morita K (2004). Development and evaluation of a novel loop-mediated isothermal amplification method for rapid detection of severe acute respiratory syndrome coronavirus. J Clin Microbiol.

[B5] Parida M, Posadas G, Inoue S, Hasebe F, Morita K (2004). Real-time reverse transcription loop-mediated isothermal amplification for rapid detection of West Nile virus. J Clin Microbiol.

[B6] Hirayama H, Kageyama S, Moriyasu S, Sawai K, Onoe S, Takahashi Y, Katagiri S, Toen K, Watanabe K, Notomi T, Yamashina H, Matsuzaki S, Minamihashi A (2004). Rapid sexing of bovine preimplantation embryos using loop-mediated isothermal amplification. Theriogenology.

[B7] Iwasaki M, Yonekawa T, Otsuka K, Suzuki W, Nagamine K, Hase T, Tasumi K, Horigome T, Notomi T, Kanda H (2003). Validation of the Loop-mediated Isothermal Amplification Method for Single Nucleotide Polymorphism Genotyping with Whole Blood. Genome Letters.

[B8] Mori Y, Kitao M, Tomita N, Notomi T (2004). Real-time turbidimetry of LAMP reaction for quantifying template DNA. J Biochem Biophys Methods.

[B9] Mori Y, Nagamine K, Tomita N, Notomi T (2001). Detection of loop-mediated isothermal amplification reaction by turbidity derived from magnesium pyrophosphate formation. Biochem Biophys Res Commun.

[B10] Osland A, Kleppe K (1977). Polyamine induced aggregation of DNA. Nucleic Acids Res.

[B11] Cordes RM, Sims WB, Glatz CE (1990). Precipitation of Nucleic Acids with Poly (ethyleneimine). Biotechnol Prog.

[B12] Lemkine GF, Demeneix BA (2001). Polyethylenimines for in vitro gene delivery. Curr Opin Mol Ther.

[B13] Bousif O, Lezoualc'h F, Zanta MA, Mergny MD, Scherman D, Demeneix B, Behr JP (1995). A versatile vector for gene and oligonucleotide transfer into cells in culture and in vivo: polyethylenimine. Proc Natl Acad Sci USA.

[B14] Raspaud E, Olvera de la Cruz M, Sikorav JL, Livolant F (1998). Precipitation of DNA by Polyamines: A Polyelectrolyte Behavior. Biophysical Journal.

[B15] Kunath K, von Harpe A, Fischer D, Petersen H, Bickel U, Voigt K, Kissel T (2003). Low-molecular-weight Polyethylenimine as a non-viral vector for DNA delivery: comparison of physicochemical properties, transfection efficiency and in vivo distribution with high-molecular-weight polyethylenimine. J Control Release.

[B16] Burns MA (2002). Everyone's a (Future) Chemist. Science.

